# Disease phenotype prediction in multiple sclerosis

**DOI:** 10.1016/j.isci.2023.106906

**Published:** 2023-05-19

**Authors:** Stephanie Herman, Staffan Arvidsson McShane, Christina Zjukovskaja, Payam Emami Khoonsari, Anders Svenningsson, Joachim Burman, Ola Spjuth, Kim Kultima

**Affiliations:** 1 Department of Medical Sciences, Clinical Chemistry, Uppsala University, Uppsala, Sweden; 2 Department of Pharmaceutical Biosciences, Uppsala University, Uppsala, Sweden; 3 Department of Neuroscience, Neurology, Uppsala University, Uppsala, Sweden; 4 Department of Biochemistry and Biophysics, National Bioinformatics Infrastructure Sweden, Science for Life Laboratory, Stockholm University, Box 1031, 17121 Solna, Sweden; 5 Department of Clinical Sciences, Danderyd Hospital, Karolinska Institutet, Stockholm, Sweden

**Keywords:** Health technology, Neuroscience

## Abstract

Progressive multiple sclerosis (PMS) is currently diagnosed retrospectively. Here, we work toward a set of biomarkers that could assist in early diagnosis of PMS. A selection of cerebrospinal fluid metabolites (n = 15) was shown to differentiate between PMS and its preceding phenotype in an independent cohort (AUC = 0.93). Complementing the classifier with conformal prediction showed that highly confident predictions could be made, and that three out of eight patients developing PMS within three years of sample collection were predicted as PMS at that time point. Finally, this methodology was applied to PMS patients as part of a clinical trial for intrathecal treatment with rituximab. The methodology showed that 68% of the patients decreased their similarity to the PMS phenotype one year after treatment. In conclusion, the inclusion of confidence predictors contributes with more information compared to traditional machine learning, and this information is relevant for disease monitoring.

## Highlights

• Fifteen metabolic features enable differentiation between multiple sclerosis phenotypes
• Conformal Prediction (CP) aid in predicting transitional phase of RRMS to PMS
• CP provides new insights for disease monitoring in a clinical trial of rituximab

## Graphical abstract

**Figure:**
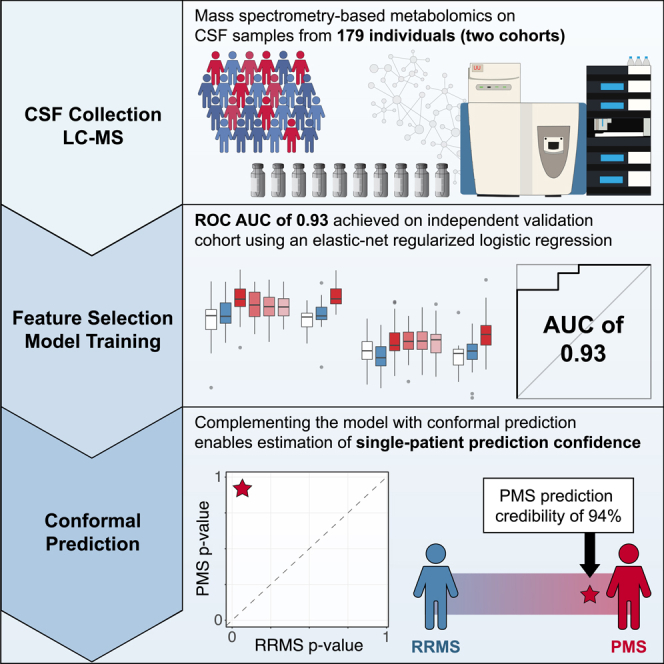

## Introduction

Multiple sclerosis (MS) is an inflammatory disease leading to demyelination, axonal injury and neurodegeneration. At onset, the most common disease course is relapsing-remitting (RRMS), when pathophysiology is dominated by formation of new inflammatory lesions in the brain and spinal cord, leading to demyelination and the clinical phenomenon of relapses. Gradually, a shift toward progressive MS (PMS) occurs in most patients if no treatment is instituted.^1^ At this stage, newly emerging lesions are rare. Instead, lesions are gradually expanding and neurodegeneration is abundant with increasing disability.^2^ With time, an increasing number of patients will be diagnosed with PMS, but the interindividual variation in reaching this point is considerable.

The clinical syndrome of PMS is based on accumulation of disability. To date, there are no neurophysiological or fluid biomarkers to support the early diagnosis of PMS.^3^ As a consequence, PMS is diagnosed in retrospect when neurodegeneration has been ongoing for years. Early identification of patients eventually fulfilling the criteria of PMS, enabling intervention, would be a valuable addition to the armamentarium of clinical practitioners.^2^ Since the transition from RRMS to PMS is believed to be a gradual process, where the underlying pathophysiologies of RRMS and PMS are mixed during a transitional period, it is challenging to develop a binary classifier.^4^ Therefore, the possibility to monitor a patient’s position in the spectrum between RRMS and PMS would aid in the clinical diagnostic procedure for PMS and would be a powerful tool in clinical trials for future PMS treatments.

Metabolomics is a comprehensive profiling of the molecular network of low-weight molecules. These metabolites essentially correspond to the intermediate and end products of ongoing physiological processes. There has been a growing interest in using metabolomics technologies to study pathophysiology in MS and for biomarker discovery. Serum-based studies in MS have demonstrated that metabolomic signatures can be associated with more severe disease courses^5^ and that kynurenine-pathway related metabolites can be used to differentiate between clinical phenotypes of MS,^6^ validated in independent cohorts. Recent studies have focused on blood to diagnose MS,^7^^,^^8^^,^^9^^,^^10^ to distinguish between RRMS and PMS,^11^^,^^12^ and several have studied MS in the metabolome of cerebrospinal fluid (CSF),^12^^,^^13^^,^^14^^,^^15^^,^^16^^,^^17^^,^^18^^,^^19^^,^^20^ which has been reviewed by Bhargava and Anthony.^21^

Artificial intelligence and machine learning (ML) have opened up for interpretation of complex data that would not be possible with traditional statistics. Machine learning has previously been successfully applied in MS diagnostics to distinguish MS from non-MS subjects^9^^,^^10^^,^^14^^,^^15^^,^^16^^,^^22^ and to differentiate MS phenotypes.^6^^,^^11^^,^^12^^,^^19^^,^^20^^,^^22^ The performance of models is typically based on predictions of held-out data that was not used in the creation of the model and reported as, for example, area under the curve (AUC) or error rate. Although such methods are widely accepted for reporting average model performance, they do not convey the confidence in individual predictions and have led to discussions and ad-hoc methods for determining a model’s applicability domain.^23^

Conformal prediction (CP) is a framework for complementing single-valued predictions from standard ML classifiers with a valid measure of their confidence.^24^^,^^25^ Many commonly used algorithms such as logistic regression, random forest and support vector machines can output probability estimates for their predictions. These estimates are however not guaranteed to be accurate and often require further calibration to be valid, something that is often overlooked.^26^ Complementing an ML classifier with the CP framework guarantees that the generated predictions and their confidences are well-calibrated.^24^^,^^27^^,^^28^ The confidence is on a per-prediction basis and can be thought of as a similarity measure between the evaluated patient and the diagnostic class in the predictive space. As an example, any traditional biomarker used in clinical practice provides a point estimate that needs to be compared to a reference interval, commonly calibrated by characterizing the variation amongst healthy subjects. Similarly, CP puts the evaluated patient in perspective to the known cases from each phenotype, producing a measure of its phenotypic similarity. In contrast to many other measures of applicability domain, the validity of CP is based on proven mathematical theory.^24^

In this study, we investigate an alternative approach to evaluate the transition to PMS. By finding a limited number (n = 15) of discriminatory CSF metabolites that are measured using high-resolution mass spectrometry, a classifier was trained and validated in an independent cohort (AUC = 0.93), and complemented with CP. As PMS patients naturally are older than RRMS patients, the full set of measured metabolites were first filtered to remove those displaying correlation to age, estimated based on healthy subjects. The final selection of metabolites was then achieved by training an elastic-net regularized classifier model, and keeping metabolites having a non-zero coefficient in the model. Finally, we demonstrate that this methodology can be used to monitor patients transitioning to the PMS phenotype and to monitor longitudinal biochemical effects of a treatment in a clinical trial in PMS.

## Results

### Subjects

#### Cohort 1

Subjects (n = 123) were recruited from the Department of Neurology at Uppsala University Hospital and Norrlands University Hospital. Thirty-nine of these patients were diagnosed with RRMS, 35 with PMS (8 primary and 27 secondary PMS) and 49 were age- and sex-matched healthy donors, Table 1. An experienced board-certified neurologist confirmed the diagnosis according to the revised McDonald and determined the course according to the revised Lublin criteria.^29^^,^^30^ The transition between RRMS and PMS was diagnosed according to the Lorscheider criteria.^31^ Twenty-two of the 35 PMS patients were part of the Intrathecal Treatment Trial in PMS (ITT-PMS), a controlled clinical trial designed to evaluate the effect of intrathecal treatment with rituximab,^32^^,^^33^ and registered with the EU Clinical Trial Register (EudraCT; 2008-002626-11 and 2012-000721-53) and ClinicalTrial.Gov (NCT01719159). The trial was registered 01/11/2012. The ITT-PMS study was a multicenter, prospective, open-label phase 1b trial, where participants were recruited between June 27, 2009, and May 11, 2015. Inclusion criteria included age between 18 and 65 years with a confirmed diagnosis of PMS within the last three years. A documented progression of neurologic symptoms over the previous two years was required, as well as an Expanded Disability Status Scale (EDSS) grading between 4.0 and 7.0. One patient with an EDSS score of 7.5 was included because one arm remained fully functional. Participants were required to be no longer eligible for conventional therapies, according to clinical practice. These patients were followed over a year after given treatment, donating a baseline CSF sample before treatment, and then again at three-, six-, and twelve-months after treatment. Clinical assessments at baseline, six- and twelve-months follow-up were collected and curated for 16 of the trial participants (Table 2), covering fatigue (fatigue scale for motor and cognitive function; FSMC), walking speed (6-min walk test and 25-foot walk test), and cognitive (symbol digit modality test; SDMT) and motoric function (9-hole peg test).^32^ The EDSS was used to assess overall disability.

**Table 1:** Clinical and demographic data for the two cohorts

*Cohort 1*	Controls	RRMS	PMS
*n*	49	39	35
Age, mean(±SD)	35(±15.6)	33(±9.10)	50(±9.53)
Female/Male	28/21	27/12	22/13
EDSS, median(range)	N/A	2.0(0.0–4.0)	6(2–7.5)
Disease duration in months, mean(±SD)	N/A	37(±64)	184(±100)
Transitioned, *n*	N/A	4	N/A
Ongoing treatment, *n*	N/A	3	1

***Cohort 2***			

*n*	10	30	16
Age, mean(±SD)	39(±13.1)	39(±10.6)	58(±9.3)
Female/Male	6/4	21/9	10/6
EDSS, median(range)	N/A	2.0(0–7.5)	5.5(3.0–7.5)
Disease duration in months, mean(±SD)	N/A	115(±103.8)	281(±128.3)
Transitioned, *n*	N/A	4	N/A
Ongoing treatment, *n*	N/A	15	1

Four RRMS patients in each cohort developed PMS within three years after sample collection.

N/A: not applicable; PMS: progressive multiple sclerosis; RRMS: relapsing-remitting multiple sclerosis; SD: standard deviation.

**Table 2:** Clinical assessment of the PMS patients in Cohort 1 part of the ITT-PMS, stratified on time point

	Baseline	6 months	12 months
EDSS, median(range)	6.3(4.0–7.0)	6.0(4.0–7.0)	6.0(4.0–7.0)
SDMT, median(range)	48.5(22.0–65.0)	52.0(19.0–65.0)	52.5(24.0–67.0)
6-MWT in m/s, median(range)^a^	0.73(0.19–1.23)	0.53(0.15–1.42)	0.59(0.17–1.31)
25-FWT in m/s, median(range)^a^	1.0(0.47–1.62)	0.92(0.29–1.73)	0.96(0.29–1.81)
Cognitive FSCM, median(range)	32.0(12.0–42.0)	32.0(16.0–47.0)	34.5(18.0–45.0)
Motoric FSCM, median(range)	41.5(19.0–50.0)	38.5(26.0–49.0)	41.5(26.0–49.0)
9-HPT (dominant), median(range)	26.2(16.7–53.7)	26.2(17.2–51.2)	26.9(16.4–49.2)
9-HPT (non- dominant), median(range)	27.3(19.3–50.1)	26.1(18.8–47.3)	25.5(18.3–44.7)

16 of the treated patients also received a clinical assessment at an extended follow-up 32–62 months after treatment, Figure S1.

a not full coverage. 25-FWT: 25-ft talk test; 6-MWT: 6-min walk test; 9-HPT: 9-hole peg test; EDSS: expanded disability status scale; FSCM: fatigue scale for motor and cognitive function; SDMT: symbol digit modalities test.

#### Cohort 2

For independent validation we used a previously described and publicly available cohort (Cohort 2),^19^^,^^20^ consisting of 56 subjects, of which 30 were diagnosed with RRMS, 16 with PMS (all secondary PMS) and 10 were controls with other non-inflammatory neurological diseases (e.g. idiopathic intracranial hypertension or thunderclap headache), Table 1. The data is accessible through MetaboLights: MTBLS558.

### Fifteen metabolic features were selected as discriminatory in cohort 1

**Figure 1:**
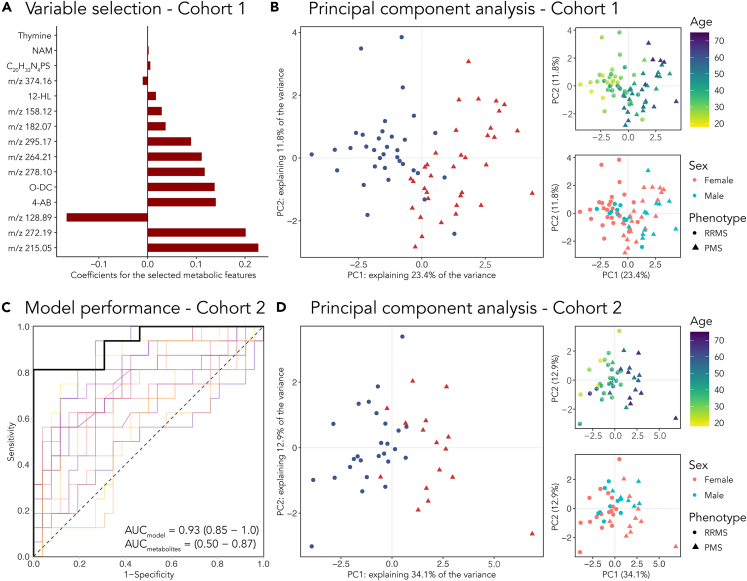
The selected metabolic features and their descriminating ability The 15 selected metabolic features, where (A) shows the penalized coefficients of the selected features in the regularized model. (B) Principal component analysis (PCA) of the selected features in cohort 1, where PMS (*red triangles*) and RRMS (*blue circles*) patients are well separated. The PCA score plot has been color-coded according to age and sex to the right. (C) The model performance when predicting the MS phenotypes of the patients in cohort 2, depicted in an ROC with a corresponding AUC of 0.93 (95% confidence interval of 0.85–1.0), which was an improvement over the best performing individual feature (AUC = 0.87). (D) PCA of the selected features in cohort 2, where a clear separation can be seen between the RRMS and PMS patients over principal component 1 (PC1). PC loadings for panel B and D can be found in Figure S3 Abbreviations used in panel A; 4-AB: 4-acetamidobutanoate, 12-HL: 12-hydroxylaurate, O-DC: O-Decanoyl-L-carnitine, NAM: nicotinamide.

In total, 498 metabolic features were successfully matched between the cohorts, with a 90% coverage and no age association in healthy controls (see section Quantification in Methods). To extract a limited set of discriminatory metabolic features that could distinguish the PMS from the RRMS patients, an elastic-net regularized logistic regression model was trained on cohort 1. The regularized model shrunk the coefficients of all but 15 metabolic features to zero, Figure 1A and Table 3. To visualize these selected metabolic features in cohort 1, a principal component analysis (PCA) was performed that revealed a statistically significant separation (R = 41, p value = 0.001) between the RRMS and PMS patients in cohort 1, Figure 1B. Owing to the documented age difference in RRMS and PMS patients in cohort 1 (Table 1), there was statistically significant separation over age (R = 0.19, p value = 0.016), but no separation between sex (R = −0.0072, p value = 0.532).

**Table 3:** The selected subset of metabolic features and the result from the statistical tests of the features in isolation in cohort 1/cohort 2 (1/2)

m/z (Da)	Identity	KEGG ID	Identity source	AUC (cohort 2)	log_2_ FC (½)	log_2_ FC p value (½)	p value sex (½)
215.05	–	–	–	0.87	0.60/0.74	∗∗∗/∗∗∗	./-
272.19	–	–	–	0.73	0.38/0.37	∗∗∗/∗∗	∗∗/-
128.89^a^	–	–	–	0.50	-0.19/-0.01	∗∗/-	./-
146.08	4-Acetamidobutanoate	C02946	Library (MS/MS)	0.80	0.26/0.40	∗∗∗/∗∗∗	-/-
316.25	O-Decanoyl-L-carnitine	C03299	MetFrag	0.62	0.57/0.10	∗∗∗/-	-/-
278.10	–	–	–	0.83	0.28/0.49	∗∗∗/∗∗∗	-/-
264.21	–	–	–	0.74	0.64/0.48	∗∗∗/∗∗	-/-
295.17	–	–	–	0.73	0.48/0.10	∗∗/-	-/-
182.07	–	–	–	0.72	0.36/0.47	∗∗/∗∗	-/-
158.12	–	–	–	0.76	0.54/0.76	∗∗/∗∗	-/∗
234.21	12-Hydroxylaurate	C08317	In silico	0.57	0.46/-0.02	∗∗/-	-/-
374.16	–	–	–	0.64	-0.18/-0.13	∗/.	-/∗
393.22	C_20_H_33_N_4_PS	–	Compound discoverer	0.58	0.16/0.03	∗∗/-	-/-
123.06	Nicotinamide	C00153	Library (MS)	0.54	0.59/0.01	∗∗/-	-/-
127.05	Thymine	C00178	Library (MS/MS)	0.85	0.19/0.51	∗/∗∗∗	-/-

The log_2_ fold changes (FC) indicate the difference between the PMS and RRMS patients, where a positive value indicates higher levels in the PMS patients. Exact p values can be found in Table S1.

Significance levels: ‘-’ p value >0.1, ‘.’ p value <0.1, ‘∗’ p value <0.05, ‘∗∗’ p value <0.01, ‘∗∗∗’ p value <0.001. FC: fold change. Library (MS): identified with the in-house library based on m/z and retention time. Library (MS/MS): identified with the in-house library based on m/z, retention time and fragmentation pattern.

a Negatively charged ion.

### The selection of metabolites could predict the MS phenotypes of cohort 2

To evaluate the generalization power of these results, the model was used to predict the MS phenotypes of the patients in cohort 2. The predictions generated a receiver operating characteristic (ROC) curve with an AUC of 0.93, better than any of the single metabolic features in isolation (which AUC performances ranged between 0.50 and 0.87), Figure 1C and Table 3. A PCA was performed on these 15 metabolic features in cohort 2, revealing a statistically significant separation (R = 0.36, p value = 0.001) between the RRMS and PMS patients over PC1, Figure 1D. Similarly, as for cohort 1, there was a close to significant separation over age (R = 0.16, p value = 0.123), but not between sex (R = −0.030, p value = 0.668). Projections of the transitioning patients are shown in Figure S2 and loadings for PC1 and PC2 in Figure S3.

### Conformal prediction provides confidence in individual patient predictions

**Figure 2:**
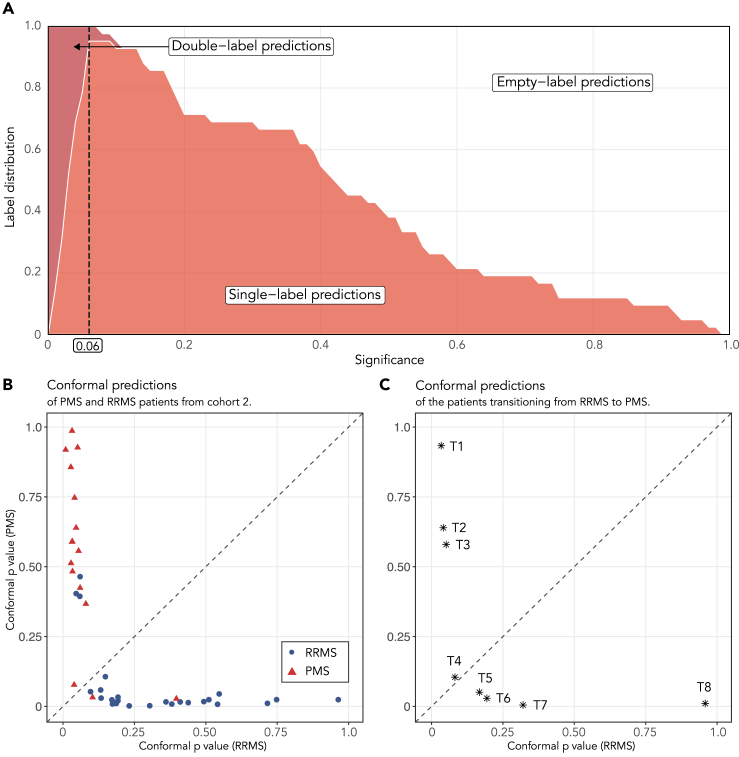
Conformal prediction performed on the extracted biochemical signature The model was trained on cohort 1. (A) The label distribution of empty, single, and double-label predictions at various significance levels, where the significance level, normally denoted ***ε***, corresponds to 1-confidence. The peak in single-label predictions, i.e., where the conformal predictor generates the most single-label predictions, occurs at a 94% confidence level (6% significance). The corresponding calibration curve can be found in Figure S4. (B) Conformal predictions of the RRMS (*blue circles*) and PMS (*red triangles*) patients from cohort 2. (C) Conformal predictions of the eight transitioning patients (T1-T8) represented as *black stars*. Utilizing the significance threshold of 6% from (A), three of the transitioning patients are predicted as PMS with single-label predictions (T1-T3), four as RRMS with single-label predictions (T5-T8) and one patient (T4) would receive a double-label prediction, see Table S3 for further details.

To investigate how CP can supply individual patient predictions with estimates of their confidence, a CP model was built on the selected metabolic features in cohort 1 (see section Conformal Prediction in Methods for further details). The model was then used to evaluate all patients in the independent validation cohort 2, Figure 2. Using a significance threshold of 6%, the lowest significance level where the single-label predictions peak (Figure 2A), would enforce a 94% confidence in the predictions. At this significance level 88% of the patients were correctly classified — 83% with single-label predictions and 5% with double-label predictions (i.e., when the model cannot predict a single class, and assigns patients to both RRMS and PMS) and 12% were incorrectly classified with single-label predictions. The acquired accuracy (88%) thus diverged slightly from the expected 94% confidence level, which can be seen in the full calibration curves for all significance levels, Figure S4.

### Conformal predictions of transitioning patients

To investigate the biochemical signature in the eight transitioning patients, the same CP model was used to predict their MS phenotypes, Figure 2C. Utilizing the significance threshold of 6% derived from Figure 2A, three of the transitioning patients are predicted as PMS with single-label predictions (T1-T3). All three of these patients obtained high p values for the PMS phenotype while simultaneously acquiring a low p value for the RRMS phenotype. One patient (T4) would be predicted as both phenotypes (a double-label prediction), as it displayed p values higher than the threshold for both phenotypes. Finally, four of the transitioning patients would still be predicted as RRMS with single-label predictions (T5-T8), even though two of them (T5 and T6) received quite low (<0.20) RRMS p values. The exact conformal p values for the transitioning patients as well as their predicted phenotype can be found in Table S3.

### Intrathecal rituximab affects the biochemical signature in PMS patients

**Figure 3:**
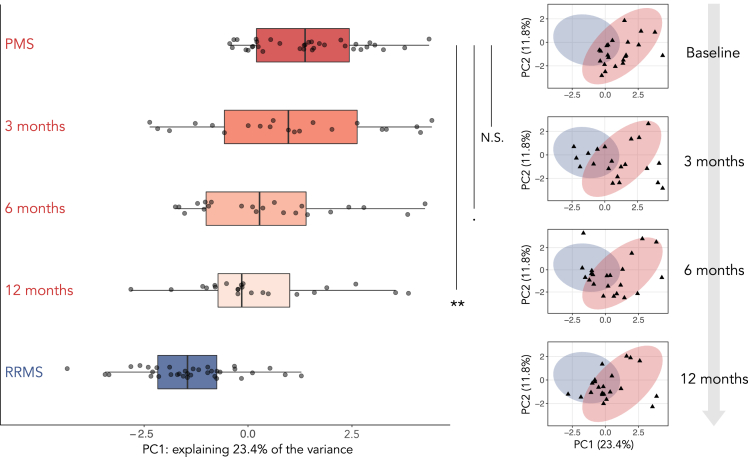
Progression of the ITT-PMS trail participants' signature over time The repeated samplings from the participants in the phase 1b clinical trial of rituximab for PMS, where the principal component (PC) 1 scores are shown as boxplots to the left and the score plot of PC1 and PC2 to the right (the treated patients are shown as *black triangles*). The boxplots visualize five summary statistics: the median, two hinges and two whiskers. A significant difference on a group-level can be seen after one year. Statistical significance is marked with the following levels: ‘N.S.’ non-significant, ‘.’ p value <0.1, ‘∗∗’ p value <0.01, and calculated by paired Wilcoxon signed-rank tests.

To investigate whether the intrathecal treatment with rituximab in the ITT-PMS trial altered the biochemical signature, the participants’ signatures at three-, six-, and twelve-months follow-up were projected into the PCA score space of cohort 1, Figure 3. With time, the participants converged biochemically toward the RRMS patients, demonstrating close to significant changes after six months (p value<0.1) and statistically significant differences at the twelve months follow-up on a group-level (p value<0.01).

### Conformal predictions of ITT-PMS participants reveal decreased similarity to the PMS phenotype after 12 months

**Figure 4:**
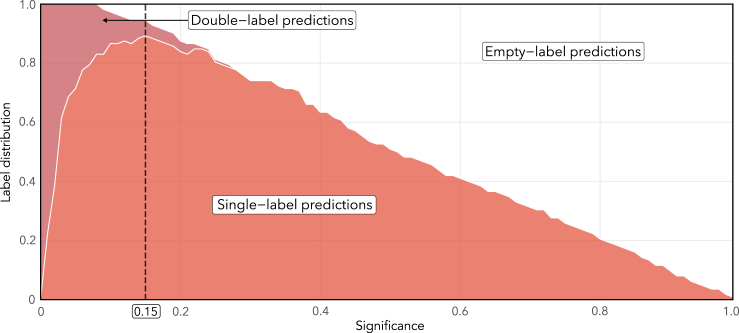
Conformal prediction analysis using both cohorts for training Analyzing the cohorts jointly and predicting patients using a leave-patient-out strategy yielded the following distribution of labels across significance levels. Only baseline samples were used for patients’ part of the ITT-PMS trial. Compared to the efficiency plot in Figure 2A, here the peak in single-label predictions both occur at a higher significance level and at a lower percentage, resulting in a less efficient model overall. The change is smoother for significance levels that are of interest, e.g., 0.05–0.1 (corresponding to 90–95% confidence), allowing us to pick any desirable level of significance in that interval. The corresponding calibration curve can be found in Figure S4.

**Figure 5:**
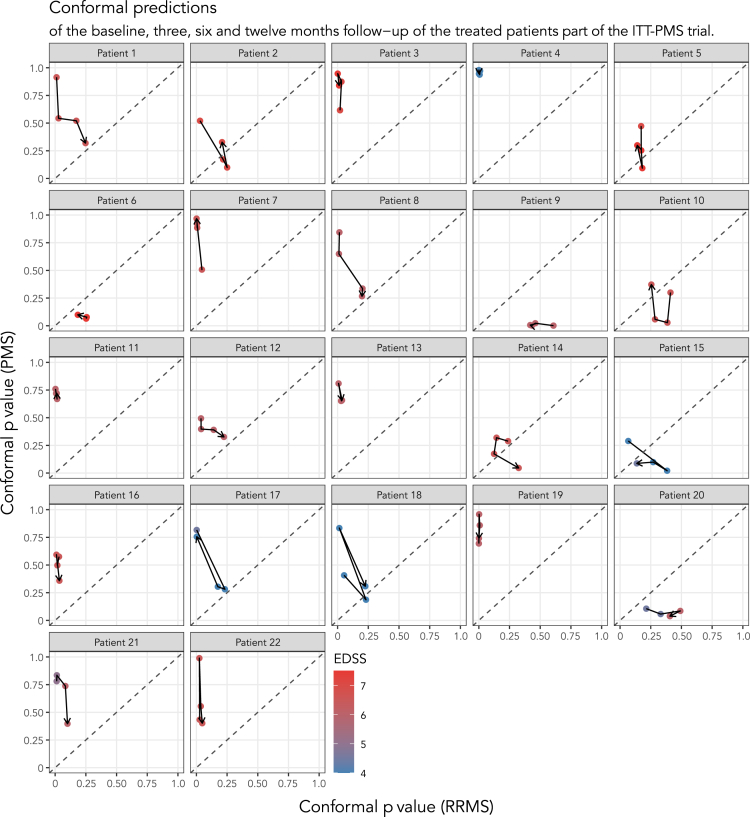
Conformal predictions of the PMS patients’ part of the ITT-PMS trial at baseline (before treatment) and at their three-, six-, and twelve-month follow-up Fourteen of the patients were classified as PMS with single-labels at baseline and seven patients received double-label predictions. Fifteen of the patients had decreased their PMS conformal p value at the twelve months follow-up. For all exact p values and predicted labels, see Table S4.

To further investigate the biochemical effect of the intrathecal treatment with rituximab, CP models were trained on both cohorts, excluding the PMS patient to be evaluated (now displaying perfect calibration, calibration curves can be found in Figure S4). The resulting efficiency of these predictions were worse than for the model trained exclusively on cohort 1 data (Figure 4), but had a less pronounced drop in percentage of single-label predictions moving from 6 to 5% significance level, allowing us to use a more conventional 5% significance level (i.e., 95% confidence). Using this significance threshold of 5% resulted in 14 patients being predicted as PMS with single-label predictions at baseline and seven patients receiving double-label predictions (Figure 5 and Table S4). One patient would be incorrectly predicted as RRMS with a single-label prediction.

At the twelve months follow-up, only nine patients received single-labeled PMS predictions, whereas eleven patients received double-label predictions and two patients were predicted as RRMS (Table S4). Overall, 68% (n = 15) of the participants had decreased their PMS conformal p values at the 12 months follow-up, indicating a decreased similarity to the PMS phenotype compared to baseline. The average difference at the twelve months follow-up to baseline is a reduction of 0.118 in the PMS conformal p values and an increase by 0.044 in the RRMS conformal p values. Extended follow-up with clinical measures from 16 of the ITT-PMS participants can be found in Figure S1.

### The selected metabolic features were associated with clinical measures

**Figure 6:**
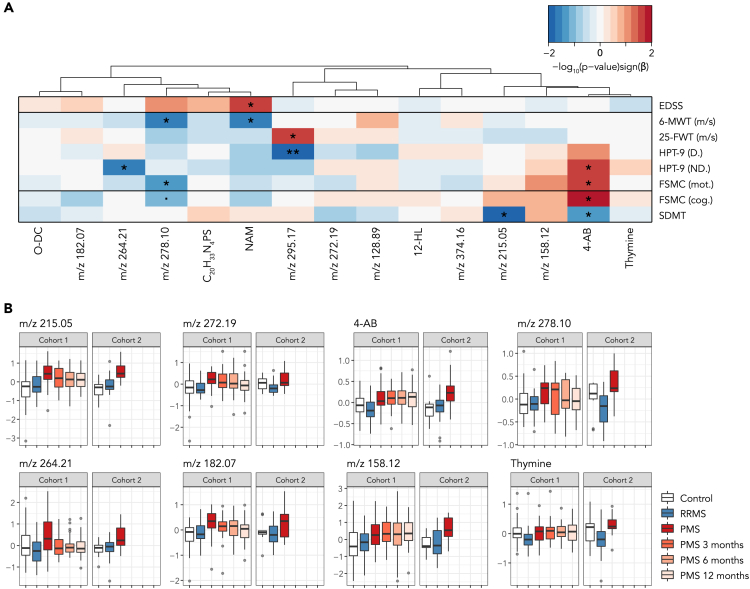
Characterisation of the selected metabolic features (A) Associations between the metabolites and eight clinical measures: 25-FWT (m/s), 6-MWT (m/s), 9-HPT of the dominant (D) and non-dominant (ND) hand, EDSS, the SDMT and the cognitive and motor scores of FSMC. Statistical significance is marked with asterisks: ‘.’ p value<0.1, ‘∗’ p value<0.05 and ‘∗∗’ p value<0.01, and calculated based on multilevel linear models. To estimate the strength and direction of the associations, the sign of each coefficient β was multiplied by the corresponding -log_10_ transformed p value. Positive associations are marked in *red*, and negative in *blue*. Hierarchical clustering analysis was performed using the Spearman’s correlation as a similarity measure. (B) The eight metabolic features that showed statistically significant (p value<0.05) differences between RRMS and PMS patients in both cohorts. The longitudinal measures at the three-, six- and twelve-months follow-up from the ITT-PMS trial are shown in cohort 1 as boxplots. The boxplots visualize five summary statistics: the median, two hinges and two whiskers. 4-AB: 4-acetamidobutanoate, 12-HL: 12-hydroxylaurate, O-DC: O-Decanoyl-L-carnitine, NAM: nicotinamide, 25-FWT: 25-ft talk test; 6-MWT: 6-min walk test; 9-HPT: 9-hole peg test; EDSS: expanded disability status scale; FSCM: fatigue scale for motor and cognitive function; SDMT: symbol digit modalities test.

To examine if the selected metabolic features were related to clinical measures, associations were investigated using multilevel models and the longitudinal measures from the ITT-PMS trial. Six metabolic features were found to be statistically significantly (p value<0.05) associated with one or several clinical measures (Figure 6A). 4-Acetamidobutanoate was significantly associated with the cognitive and motor scores of the fatigue scale for motor and cognitive function (FSMC), the 9-hole peg test (9-HPT) of the non-dominant hand and the symbol digit modalities test (SDMT), whereas nicotinamide was associated with EDSS and walking speed. Based on univariate statistics, the metabolites 4-acetamidobutanoate, thymine and the unidentified metabolic features with *m*/*z* of 215.05, 272.19, 278.10, 264.21, 182.07, and 158.12 had statistically significantly higher concentrations in PMS compared to RRMS patients in both cohorts, (Figures 6B and Table 3). None of the metabolic features showed any statistically significant differences between patients with an ongoing treatment versus those without.

## Discussion

Progressive MS is currently diagnosed in retrospect, mainly because of the vague functional definition and current diagnostic strategy of PMS.^30^^,^^31^ In the present study, we investigated an alternative approach to assist the diagnosis of PMS in the future. A selection of CSF metabolic features that could discriminate between RRMS and PMS patients (validated in an independent cohort, AUC = 0.93) were used to create a CP classification model that correctly classified 88% of MS patients from the independent cohort, at a prediction confidence threshold of 94%. We further demonstrated how this methodology could be used to investigate the biochemical signature of an RRMS patient that is transitioning to the PMS phenotype within two-to-three years, and how it could be used to monitor longitudinal changes in patients who are participating in a clinical trial.

Today the PMS diagnosis is typically given after a careful examination of the patients’ historical disease course and the rate of permanent disability accumulation. However, because the brain has the ability to compensate for neuronal loss, it is not until these mechanisms are exhausted that the signs will manifest.^34^^,^^35^ As a consequence, the diagnosis is given years after the pathophysiological and biochemical changes have been developed.

The 15 selected metabolic features were extracted in cohort 1 using a regularized logistic regression model. Using the model to predict the MS phenotypes of patients in the independent validation cohort 2 resulted in an AUC of 0.93. Furthermore, the CP model that was constructed on these 15 metabolic features and trained on cohort 1 was able to correctly classify 88% of the patients in cohort 2, of which 83% were predicted with single-label predictions, at a 94% prediction confidence threshold. Previously, serum metabolites from the kynurenine pathway have been able to correctly classify 83% of individuals in an independent cohort of RRMS patients, secondary PMS patients and healthy control subjects.^6^ Cerebrospinal fluid metabolites have also been shown to be able to distinguish PMS phenotypes from RRMS, but none of these predictive accuracies have been validated in independent cohorts,^12^^,^^19^ making it difficult to compare previous results to results herein.

We hypothesized that CP could convey a phenotype prediction confidence in individual predictions that could be used to detect biochemical deviations from an RRMS pathology and thus assist in detecting the initiation of a transitional event. The study cohorts included eight RRMS patients that were clinically diagnosed with PMS two-to-three years after donating a sample. Therefore, the MS phenotypes of these patients could be considered uncertain and they were instead referred to as transitioning patients and were held-out as an external patient group. Predicting their MS phenotypes at the 94% confidence threshold, resulted in four patients being predicted as RRMS with single-labels, one patient receiving a double-label prediction and three patients that were predicted as PMS with single-labels. As the transition from RRMS to PMS is believed to be a gradual process, where the underlying pathophysiologies of RRMS and PMS are mixed during a transitional period,^4^ we believe that enforcing a binary classifier with CP opens up opportunities to detect patients whose transitional period is in an early stage.

In the present study, CSF samples from patients part of the ITT-PMS clinical trial were analyzed both from their baseline (before treatment) and their follow-up samples at three-, six-, and twelve-months. This provided an opportunity to investigate if any changes in the biochemical signature of MS could be detected. Based on the biochemical signature of the participants’, a statistically significant difference at the twelve months follow-up was found on a group level. Using CP to evaluate the confidence in their phenotype predictions, 14 of the patients were predicted as PMS with single-labels and seven patients received double-label predictions at a 95% prediction confidence threshold before treatment. At the twelve-month follow-up after treatment, five of the single-label PMS predictions were instead predicted as double-labels (RRMS and PMS), and 68% of the patients had decreased their PMS conformal p values and thus their similarity to the PMS phenotype. Longitudinal changes in the biochemical signature demonstrate that the methodology is able to detect dynamic changes and features of MS, which is important in continuous monitoring and evaluation of, e.g., therapeutic interventions. Hence, we argue that utilizing a similar, but more mature, methodology as an endpoint in clinical trials for PMS could be used to detect changes occurring in individual patients.

The biochemical signature consisted of 15 metabolic features, of which eight were statistically significantly altered between RRMS and PMS patients in both cohorts. 4-Acetamidobutanoate was significantly increased in PMS patients compared with RRMS patients in both cohorts. We have previously shown that 4-acetamidobutanoate is correlated with neurodegeneration in the spinal cord and third ventricle as well as with the disease duration, while showing no association with age.^19^ Here, we could show that 4-acetamidobutanoate was also associated with the clinical measures; SDMT, the 9-HTP and the cognitive and motor scores of FSMC, all part of the ITT-PMS trial. 4-Acetamidobutanoate is a precursor of gamma-aminobutyric acid (GABA) in the arginine and proline metabolism. The most well-known biosynthesis of GABA is from glutamate. However, it has been shown that a well conserved GABA synthesis pathway is being used by midbrain dopaminergic neurons. These cells synthesize GABA from putrescine, having 4-acetamidobutanoate as an intermediate product.^36^ Putrescine has been shown to be increased in the CSF of the Experimental Autoimmune Encephalomyelitis (EAE) mouse model of MS at a simulated peak of the disease.^37^^,^^38^ The evidence of 4-acetamidobutanoate’s relation to MS is growing, but further studies are required to elucidate the biological background for these observations.

Another metabolite that showed statistically significantly increased CSF levels in PMS was thymine. We have previously demonstrated that thymine was significantly associated with EDSS, disease duration and neurodegeneration in the spinal cord of MS patients,^19^ but no significant associations with the clinical measures in the ITT-PMS trial were found herein. Nucleotide metabolism has previously been noted perturbed in RRMS patients compared with healthy controls.^39^ Pyrimidine synthesis inhibitors are used in treatment of RRMS to block *de novo* pyrimidine synthesis. These inhibitors interrupt the S phase of the cell cycle in proliferating active T and B cells, limiting their reproduction and involvement in inflammatory processes,^40^^,^^41^ however none of the patients being investigated herein were being treated with such drugs. The remaining metabolites that were selected and identified have, to our knowledge, not been investigated in relation to MS. As for the metabolic features that were not identified in the current study, we hope that their inclusion will lead to their future identification and validation by either us or other groups in the field.

To implement a similar methodology in healthcare, the biomarkers would have to be carefully selected and validated in isolation as well as together, and they would have to be absolutely quantified. Absolute quantities would enable the model to make straightforward predictions of new patients. Once a patient has been successfully diagnosed by the clinical neurologist, it can be added into the growing datasets used for building the classification models. In terms of reported outputs, a conformal p value set (p[RRMS], p[PMS]) would be delivered to the clinician, conveying the patient’s biochemical status and its similarity to both phenotypes. Monitoring an RRMS patient’s similarity to the MS phenotypes would enable an early detection of deviations from the patient’s habitual state, that could potentially indicate an initiation of the PMS pathology. Furthermore, this methodology is not limited to the diagnostic procedure of MS, but could also be applied for diagnosing other diseases with similar diagnostic challenges, such as Alzheimer’s disease that typically develops from a state of mild cognitive impairment (MCI).

The CSF samples from 49 healthy control subjects in cohort 1 was an important asset in the study. As there is a natural age difference between the RRMS and PMS patients (as PMS typically follows the RRMS phase), age-dependent metabolites will be overlapping with discriminatory metabolites in the MS patients. The MS patients can therefore not be used to evaluate and exclude age-dependent metabolites, as it would risk removing disease-related metabolites. Using healthy control subjects to detect and exclude age-dependent metabolites is therefore a suitable way to resolve this issue.

### Limitations of the study

A natural limitation of the study is the limited number of subjects. This primarily originates from the fact that PMS patients constitute a minority of MS patients. For an RRMS patient that is transitioning to (secondary) PMS, a second CSF sample is seldom collected. Only when investigating and diagnosing an MS naive individual with (primary) PMS, a CSF sample is typically collected and part of the diagnosis. A limited number of subjects in a cohort will limit the statistical power. In the statistical tests of the selected metabolic features in isolation, all features were found to be statistically significantly altered between RRMS and PMS patients in cohort 1, whereas only eight were statistically significant in the independent validation cohort 2. However, the sample size of cohort 2 was smaller, which reduces statistical power.

The availability of these two independent cohorts, where one could be used for selecting the metabolic features and model construction, and the other for performance estimation, was advantageous in terms of result validation. However, using these cohorts separately, came at the cost of the calibration of the final model complemented with CP. The model that was trained on cohort 1, had a calibration curve that deviated from the expected error rate (both positively and negatively). Deviations from the expected result can either be because of small test set size or violations to the exchangeability criterion (i.e., cohort 1 and cohort 2 are not from the same underlying distribution). When evaluating the two cohorts jointly, the calibration curves instead displayed a perfectly well-calibrated model. We believe that these results can be explained by a systematic difference between the patient-samples from cohort 1 and 2, e.g., the difference in mean age and disease duration (Table 1), where patients in cohort 2 are both older and had a longer disease duration. When analyzing the cohorts jointly they instead form a joint distribution and thus display well-calibrated predictions. A strength of the CP methodology is that the level of calibration is quantified so that errors can be identified and rectified.

### Conclusions

Altogether, this study demonstrates that it is plausible to generate a condensed subset of small molecules that is able to distinguish PMS from RRMS patients and that CP can be used to generate valid individualized evaluations of the MS phenotype. This methodology holds promise in detecting transitioning patients earlier and in monitoring disease progression in individual patients. Furthermore, this methodology is not limited to MS, but could also be useful for other diseases with similar diagnostic challenges.

## STAR★Methods

### Key resources table

**Table:** 

REAGENT or RESOURCE	SOURCE	IDENTIFIER
**Deposited data**		

Cohort 1 metabolic data	This paper	https://www.ebi.ac.uk/metabolights/MTBLS1464 ; MetaboLights: MTBLS1464
Cohort 2 metabolic data	(Herman et al., 2019; 2018)^19^^,^^20^	https://www.ebi.ac.uk/metabolights/MTBLS558 ; MetaboLights: MTBLS558

**Software and algorithms**		

R statistical software v3.6.0	R Core team^42^	www.R-project.org/
Scikit learn python library version 0.23.2	(Pedregosa et al., 2012)^43^	https://scikit-learn.org/
Nonconformist commit 91fca869b7421c0658fd93590a6d84d23a96072d	(Linusson H)^44^	https://github.com/donlnz/nonconformist
Code used within this project	This paper	https://github.com/stephanieherman/MS-phenotype-prediction; https://doi.org/10.5281/zenodo.7829061

### Resource availability

#### Lead contact

Further information and requests for resources should be directed to and will be fulfilled by the lead contact, Kim Kultima (kim.kultima@medsci.uu.se).

#### Materials availability

This study did not generate new unique reagents.

### Experimental model and subject details

The study was approved by the Regional Ethical Review Board in Umeå (EPN; 2009-08-157M) and the Regional Ethical Board of Uppsala (Dnr 2015/462 and Dnr 2013/278). The study was performed in accordance with standards of good clinical practice and the principles of the Declaration of Helsinki. Oral and written information about the study was provided to all participants before written consent was obtained.

#### Cohort 1

Cohort 1 included in total 39 RRMS patients (sex = 27 female, 12 male; mean age = 33; age SD = 15.6), 35 PMS patients (sex = 22 female, 13 male; mean age = 50; age SD = 9.53) and 49 controls (sex = 28 female, 21 male; mean age = 35; age SD = 15.6). Out of the 35 PMS patients eight were diagnosed with primary PMS and 27 with secondary PMS.

#### Cohort 2

Cohort 2 included in total 30 RRMS patients (sex=21 female, 9 male; mean age=39; age SD=10.6), 16 PMS patients (sex=10 female, 6 male; mean age=58; age SD=9.3) and 10 controls (sex=6 female, 4 male; mean age=39; age SD=13.1). All PMS patients were diagnosed with secondary PMS.

### Method details

#### Sample collection

The lumbar puncture was performed through the L3/L4 or L4/L5 interspace and CSF was collected in accordance with the guidelines formed by the BioMS-eu network.^45^ In brief, CSF was collected into a polypropylene tube that was centrifuged at room temperature. The supernatant was extracted, gently mixed and aliquoted into polypropylene tubes that were stored at −80°C until analysis.

#### Metabolite extraction

Metabolite extraction of CSF metabolites and mass spectrometry analysis was done as previously described.^20^ In brief, metabolites were extracted using ice-cold methanol supplemented with a cocktail of internal standards that was added to 100 μL of CSF (thawed on ice). The extraction was done in four batches, over four days where diagnostic groups and sampling locations were balanced over the batches. After extraction, the samples were dried and reconstituted in 100 μL of 5% methanol, 0.1% formic acid and 94.9% deionized MilliQ water upon analysis. 10 μL of each sample was pooled to create a quality control (QC) sample to be injected repeatedly throughout the analysis.

#### Mass spectrometry analysis

10 μL of each sample was injected in a constrained randomized order to a Thermo Ultimate 3000 HPLC equipped with a Thermo Accucore aQ RP C18 column (100 × 2.1 mm, 2.6 μm particle size) and coupled to a Thermo Q-Exactive Orbitrap. The mass spectrometer was operated in positive and negative ion mode and resolutions were set to 70 000 at *m*/*z* 200, AGC target was 1e6 and maximum ion injection time was 250 ms. The analysis was initiated with five QC injections to condition the column and equilibrate the system, followed by two blank samples. Thereafter, to aid extraction of features originating from samples, a 2-fold serial dilution series ranging from 0.5 to 32.0 μL QC was injected. A QC and a blank injection were done every 8^th^ sample to enable performance monitoring and filtering of stable features. For improving metabolite identification, eight tandem mass spectrometry analyses in both ion modes were performed separately on batch pooled samples as well as on the global pool (the QC sample).

#### Quantification

The acquired raw data was converted to an open-source format (.mzML). Peak picking was performed using *msconvert* from ProteoWizard^46^ and preprocessing using the following pipeline within the KNIME platform.^47^ The peak-picked data was quantified by *FeatureFinderMetabo*,^48^ features were aligned using *MapAlignerPoseClustering* and linked across samples using *FeatureLinkerUnlabelledQT*.^49^ The time tolerance was set to 10 s and a 5 ppm mass deviation was allowed. The non-default parameters can be found in Table S2.

The quantified data was loaded into the statistical software environment R v3.6.0.^42^ Subsequently, contaminants were removed by (1) using the blank injections, according to our previously introduced pipeline^50^ and (2) by only keeping the metabolic features that achieved a significant (p value<0.05) Pearson correlation with the dilution series. To stabilize variance, the intensity values were log_2_ transformed. To correct for runorder effects (e.g., intensity decay), LOESS curves were fitted for each metabolite using the function “loessFit” from the R-package *limma*^51^ and a span of 0.2, which were used for normalization. Finally, the coefficient of variance (CV) for each metabolic feature was calculated on the de-logged values in the QC samples and features with a CV < 0.20 were kept. Potential sample outliers were investigated by calculating the total ion count (TIC) of each sample.

To match metabolic features across cohort 1 and 2, a window of ±2.5 ppm in mass and ±5 seconds in retention time was used, as well as an average intensity deviation less than ±1 standard deviation of the average intensity differences. Age dependence was evaluated per metabolic feature in the healthy control subjects in cohort 1 that covered the complete adult age span (18-74 years). Features that displayed a significant (p value<0.05) age association by the Pearson’s correlation were excluded. Furthermore, only features that were matched and present in at least 90% of all samples in both cohorts were kept for analysis. The remaining missing values were imputed by the average feature value and, finally, all features were centered within the cohort.

#### Metabolite identification

Metabolites were identified using an in-house library consisting of 471 characterized pure standards, where the features first were matched using a ±2.5 ppm in mass and ±5 seconds in retention time window. Matches with an available MS/MS spectrum were extracted and matched to the fragmentation pattern from the in-house library. Identities of metabolites whose MS/MS spectrum did not match with the suggested library identity were rejected.

To increase the identification rate, three additional identification strategies were used. First, an *in silico* prediction of MS/MS fragmentation spectra was done on metabolites available on the Human Metabolome Database (HMDB) using the PhenoMeNal framework.^52^ Briefly, OpenMS^53^ was used to extract metabolic features, which were imported into CAMERA^54^ to predict adducts, which further were used to derive the neutral masses of the precursors. Neutral masses and MS/MS spectra were then used in CSI:FingerID^55^ to perform the *in silico* identification. Additionally, the identification workflow for untargeted metabolomics using online databases and chemical composition prediction in the Compound discoverer software from Thermo Fisher Scientific (v. 3.1.0.305) was used as well as the *metfRag* R package^56^ with the KEGG database and an allowance of 10 ppm relative mass deviation for the database search and 20 ppm for fragment peak matching.

#### Feature selection

To extract discriminatory metabolic features between RRMS and PMS patients, cohort 1 was used for model optimization, model training and variable selection. For PMS patients part of the ITT-PMS trial, only the baseline CSF sample taken before intervention was included. An elastic-net regularized logistic regression using the *glmnet* R package^57^ with alpha set to 0.5 was trained on the RRMS and PMS patients in cohort 1, with standardized metabolic features (zero mean, unit variance). The hyperparameter lambda was determined through a balanced 7-fold cross-validation, permitting five patients from each phenotype within each fold, where a lambda of one standard error from the lambda minimizing the cross-validated deviance was chosen. The performance of the logistic regression model was evaluated by predicting the MS phenotypes of patients in cohort 2 and computing the ROC and AUC using the *roc* function from the R package *pROC*.^58^ Finally, discriminatory metabolic features were extracted and ranked according to their coefficients.

#### Feature characterization

To estimate corrected log_2_ fold changes between RRMS and PMS patients of the selected features in isolation, linear models were fitted on each feature separately in the two cohorts, with the metabolic levels as response and the MS subtype and gender as covariates. To extract corrected estimates and corresponding significance levels, the *emmeans* R package^59^ was used. To ensure that ongoing treatments were not a contributing factor, the non-parametric two-sided Mann-Whitney test was used to compare the metabolic levels in patients with an ongoing treatment *versus* those without an ongoing treatment.

To visualize the selected features in both cohorts, principal component analysis (PCA) was performed using the *prcomp* from the R package *stats*.^42^ The separations between different stratifications were evaluated using the analysis of similarity with the *anosim* function from the *vegan* R package.^60^ The results were permuted 999 times. Furthermore, the transitioning patients were projected into the model space, and to evaluate the biochemical effect of rituximab in the ITT-PMS study, the follow-up samples at three-, six- and twelve-months after treatment were also projected into the model space of cohort 1. The effects were evaluated by paired Wilcoxon signed-rank tests between the principal component 1 scores at baseline and at each follow-up time point.

Finally, to investigate potential associations between the selected metabolic features and clinical measures, multilevel linear models were trained for each combination of metabolite and clinical measure in the 16 trial participants where this information was available. The *lme* function from the R package *nlme*^61^ was used, with patient identity as a random effect, metabolic feature as response and clinical measure as explanatory variable. Time point (in months) and sex were included as covariates. To allow the association between the metabolic feature and clinical measure to vary over time, the interaction term between time and clinical measure was included. Finally, the strength of the association was estimated by multiplying the -log_10_ transformed significance (p value) of the clinical measure’s coefficient with the sign (+1 or -1) of the coefficient (indicating a positive or negative association).

#### Conformal prediction

To evaluate the extracted signature of features on a per-patient level, the CP framework was used.^24^^,^^25^^,^^62^ In short, classifying patients to the two phenotypes, RRMS and PMS, results in a p value for each phenotype. This conformal p value is based on a ranking of similarity, or *conformity*, with patients of each phenotype, a high p value indicates a high similarity to the phenotype and vice versa. Similar p values to both phenotypes indicate an equal similarity or dissimilarity to both. The user has to decide on a significance level (the percentage of accepted errors in predictions) which is then applied to the p values to yield the prediction set of an individual patient. For this binary problem, patients can have four different prediction outcomes; no class, RRMS or PMS, or both classes. CP is mathematically proven to produce valid predictions, i.e., when setting the desired confidence to 95%, the correct label is excluded in at most 5% of the predictions (deviations can however occur due to finite test sample size). The only requirement is that the exchangeability criterion is met, which all standard ML algorithms already impose. Knowing that the predictions are always valid, the main objective when using CP is to optimize the efficiency of the predictor, e.g., the fraction of predictions that are single-label, and thus ‘informative’, and/or to reduce the fraction of empty and ‘both’ predictions.

In this study, the mondrian transductive CP method was used from the python package *nonconformist*^44^ using the class *TcpClassifier* set to use class-conditional calibration. The default classification nonconformity-measure in nonconformist (*MarginErrFunc*) was used, with an underlying ML model of type *SVC* (support vector classifier) from *scikit-learn*,^43^ configured to generate probability estimates. All features were normalized in the same way as described in previous sections, i.e., normalized to zero mean and unit variance (fitted using cohort 1 and using the same parameters for cohort 2).

First, using data from all RRMS and PMS patients from cohort 1, the best kernel of the SVC was found using a grid search with four possible kernels (linear, radial basis function (RBF), sigmoid and polynomial) and a small set of kernel parameters for each kernel, concluding that the RBF kernel was the one producing the best accuracy. Then, a second grid search using 30 cost (cost of miss-classifications) and 30 gamma (RBF kernel parameter) values was done to find the final parameters used in the CP evaluation. Both evaluations were performed using the leave-one-out cross-validation strategy and optimizing for best classifier accuracy.

Then, using the obtained SVC hyperparameters, a transductive CP model was trained on the RRMS and PMS patient data from cohort 1 and used for predicting the patients in the validation cohort 2 as well as the previously excluded transitioning patients from both cohorts.

As a proof of concept, conformal p values were also computed for the treated patients’ part of the phase 1b clinical trial of rituximab for PMS. Model training was then performed using all baseline samples (RRMS and PMS patients from both cohorts), excluding the patient which was being evaluated. The repeated samples of the held-out patient were then predicted using the learned models.

### Quantification and statistical analysis

All statistical analyses were performed in the statistical software environment R v3.6.0. Quantified metabolic features were required to display a statistically significant (p value<0.05) Pearson’s correlation with the 2-fold dilution series (N=6) that was injected in the beginning of the mass spectrometry analysis. Metabolic features were also filtered on their variance in the repeatedly injected QC samples (N=27), where a coefficient of variance lower than 0.2 was used as an inclusion criterion. Finally, to remove age-dependent metabolites, Pearson’s correlation was used on the relative abundance levels in the healthy control subjects in cohort 1 (N=49), where metabolic features that displayed a p value<0.05 were deemed associated with age and excluded.

To estimate the log_2_ fold changes between RRMS and PMS patients in the two cohorts (Cohort 1 RRMS N=39 & PMS=35; Cohort 2 RRMS=30 & PMS=16), linear regression including the MS subtype and gender (for correction) as covariates was used. To extract corrected estimates and corresponding significance levels, the *emmeans* R-package was used.^59^ Furthermore, to evaluate statistical significance of separations in the PCA model space, the analysis of similarity performed using the *anosim* function from the *vegan* R-package.^60^ The results were permuted 999 times and a p value<0.05 was seen as statistically significant. The principal component 1 scores of the projected repeated measurements (N=4) of the PMS patients (N=22) from the ITT-PMS study were evaluated using a paired Wilcoxon signed-rank test. Finally, to investigate associations between the selected metabolic features and clinical measures, multilevel linear models were trained on 16 of the ITT-PMS participants for whom this data was available, using the *lme* function in the *nlme* R-package.^61^ Patient ID was used as a random effect, whereas the metabolic levels were used as response and the clinical measure as the explanatory variable. To allow the association to vary over time, the interaction term between time and the clinical measure was included in the model.

### Additional resources

Details about the ITT-PMS clinical trial from ClinicalTrail.gov (identifier NCT01719159): https://clinicaltrials.gov/ct2/show/NCT01719159.

Details about the ITT-PM clinical trial from the ECU Clinical Trial Register (identifier 2008-002626-11): https://www.clinicaltrialsregister.eu/ctr-search/trial/2008-002626-11/results.

Details about the extended follow-up of the patient’s part of the ITT-PMS clinical trial from the ECU Clinical Trial Register (identifier 2012-000721-53): https://www.clinicaltrialsregister.eu/ctr-search/trial/2012-000721-53/results.

## Data Availability

• Metabolite data from cohort 1 have been deposited at MetaboLights and are publicly available as of the date of publication. Metabolite data from cohort 2 have been deposited previously at MetaboLights. Accession numbers are listed in the key resources table. • All code has been deposited at GitHub and is publicly available as of the date of publication. DOIs are listed in the key resources table • Any additional information required to reanalyze the data reported in this paper is available from the lead contact upon request.
