# Supplementary material for: Disease phenotype prediction in multiple sclerosis
Source: iScience. 2023 May 19;26(6):106906. doi: 10.1016/j.isci.2023.106906 (PMC10275960; doi:10.1016/j.isci.2023.106906)
Supplement: Document S1. Figures S1–S4 and Tables S1–S4 [file mmc1.pdf]

iScience, Volume *26*

Supplemental information

Disease phenotype prediction in multiple sclerosis

Stephanie Herman, Staffan Arvidsson McShane, Christina Zjukovskaja, Payam Emami Khoonsari, Anders Svenningsson, Joachim Burman, Ola Spjuth, and Kim Kultima

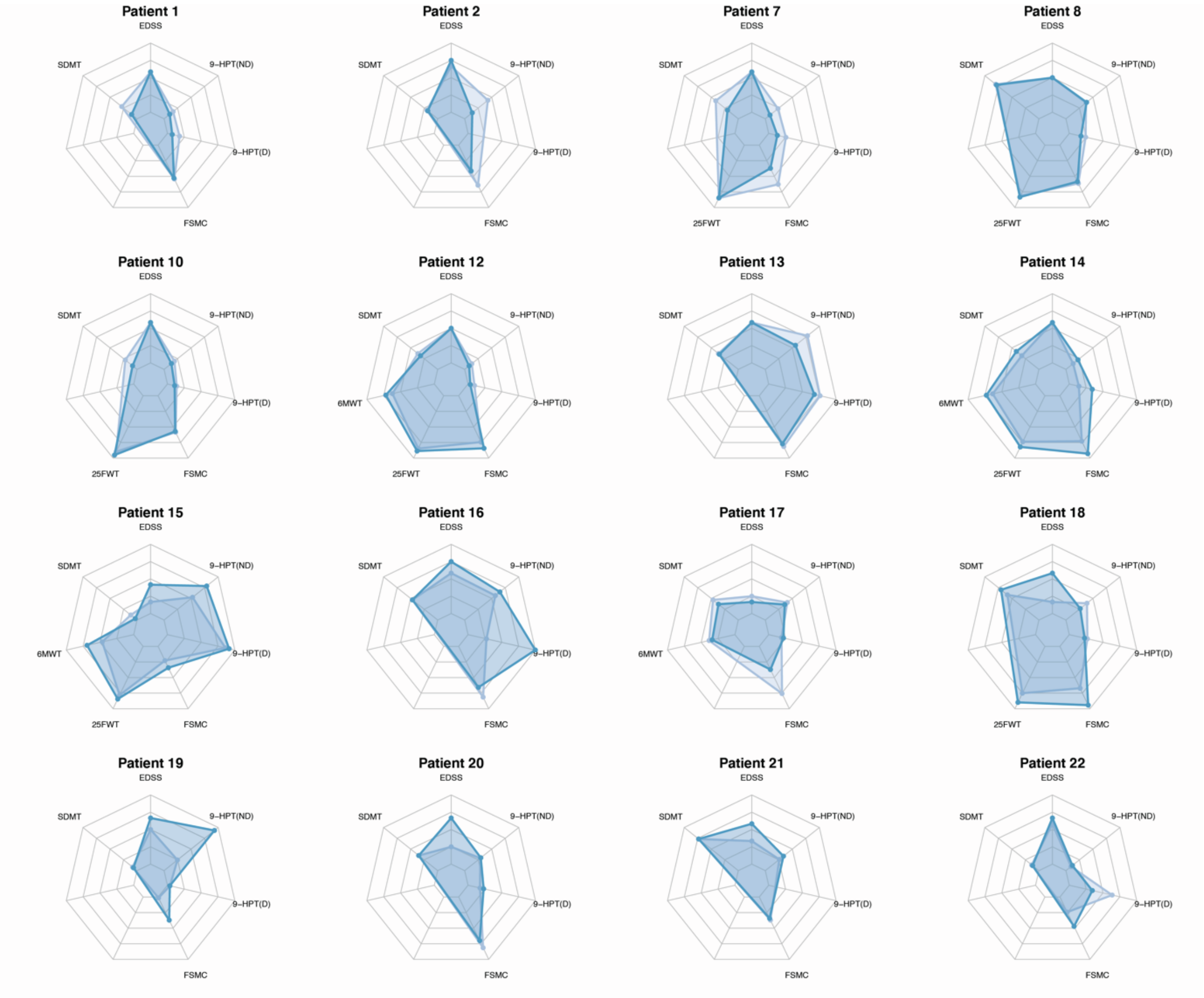

Figure S1. Extended clinical assessment of ITT-PMS patients, related to Table 2. Clinical assessments of 16 of the treated patients from the ITT-PMS trial at an extended follow-up between 32-62 months after treatment, where the *light blue* indicates the patient's state at baseline and the *dark blue* at follow-up. Names of measures that were missing in a patient have been removed. According to EDSS, eight patients had increased their disability at follow-up, which is not completely consistent with other clinical measures.

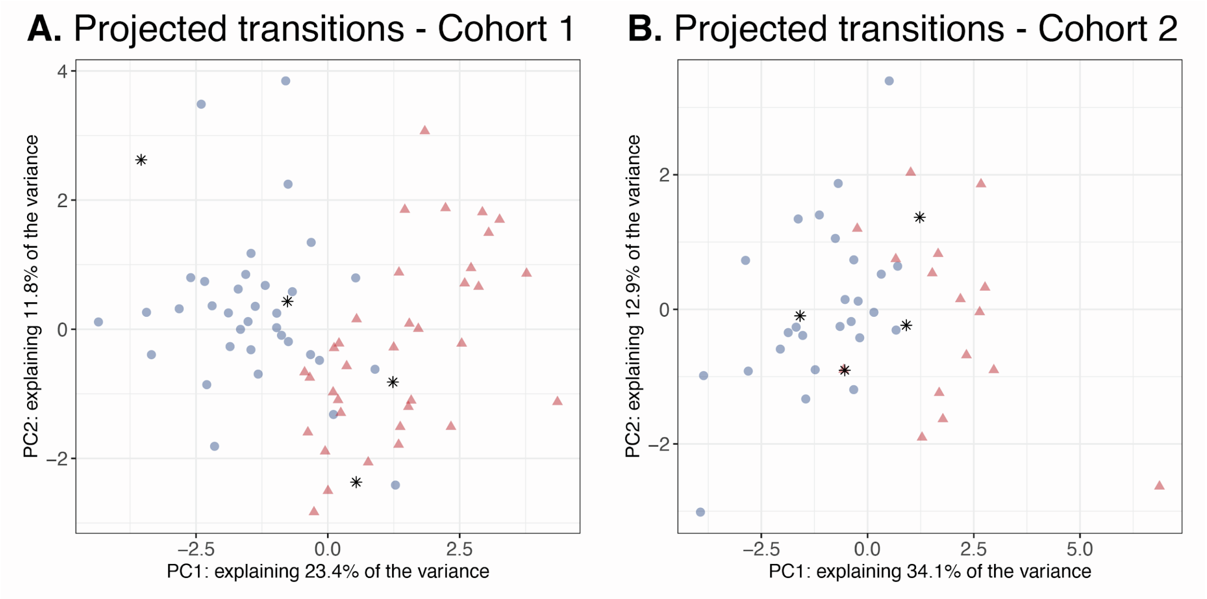

Figure S2. Projections of transitioning patients into PCA score space, related to Figure 1. Projections of the transitioning patients (*black stars*) in the principal component analysis (PCA) of cohort 1 (A) and cohort 2 (B). Patients with known subtypes are displayed in the same way as in Figure 1, with PMS (red triangles) and RRMS (blue circles)

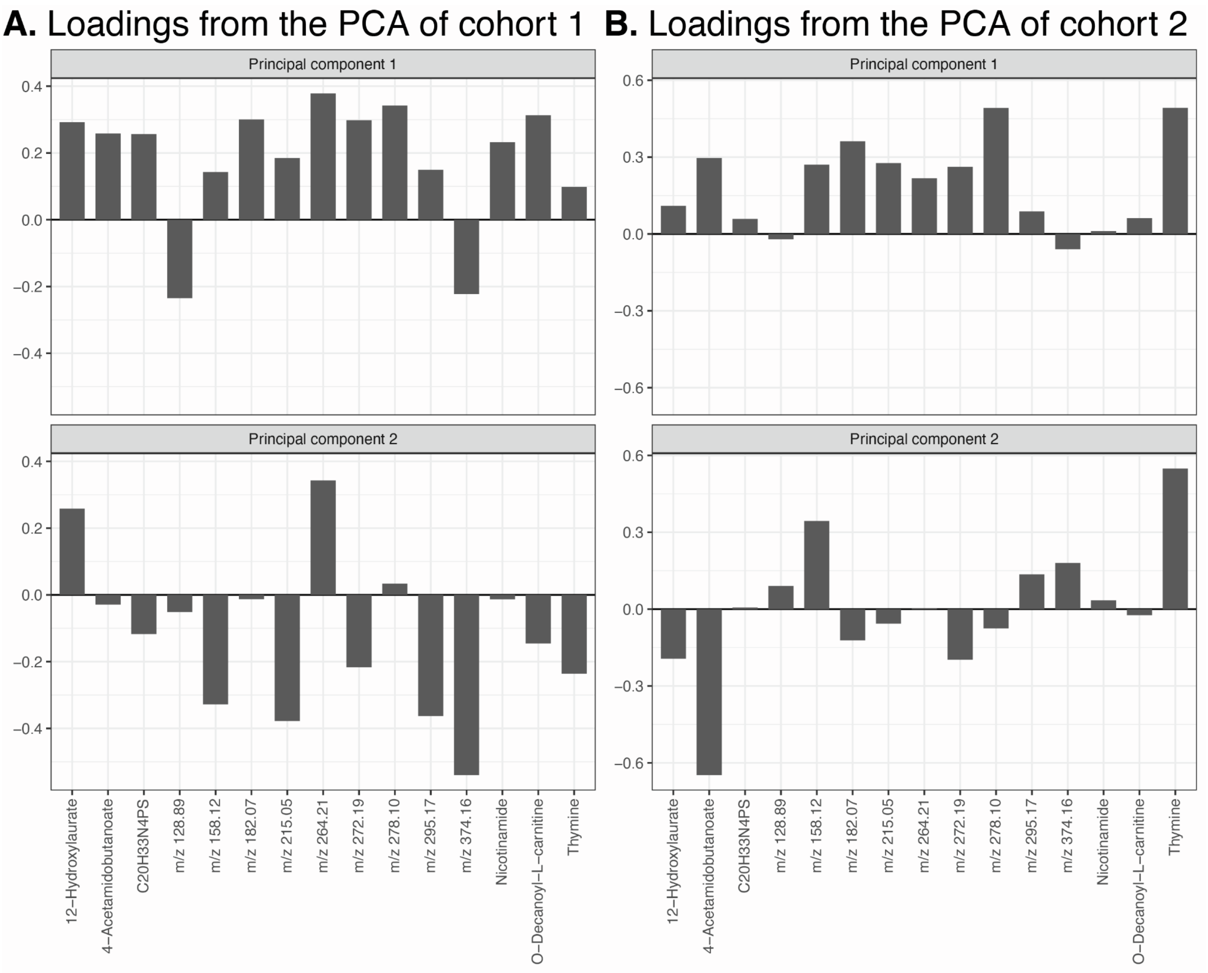

Figure S3. Loadings of principal component (PC) 1 & 2, related to Figure 1. The PC loadings for the principal component analysis (PCA) of cohort 1 (A) and cohort 2 (B) from Figure 1.

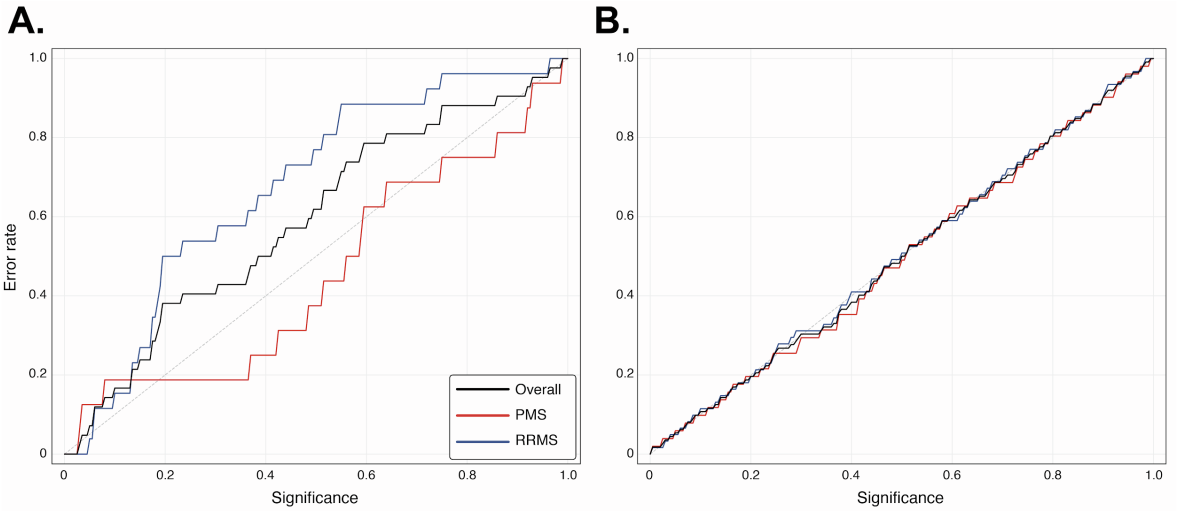

Figure S4. Calibration curves for conformal prediction models, related to Figure 2 and Figure 4. Calibration curves from the conformal prediction evaluation of the PMS and RRMS patients in cohort 2 when using cohort 1 for training the model (A) and when mixing both cohorts and using leave-one-out cross-validation (B). The overall error rate is shown in *black*, whereas the separate performances are shown in *red* (PMS) and *blue* (RRMS). For a well-calibrated (i.e., valid) model, the error rate should follow the grey dashed line (i.e., the prediction accuracy should correspond to the desired confidence level). Some deviations can however be expected when the test set is small as statistical fluctuations have a greater impact. The deviations from perfect calibration found in (A) are likely caused by violation to the exchangeability criterion, i.e., there must be a systematic difference between the two cohorts, as the calibration from modelling the cohorts jointly (B) displays a perfect calibration. However, there is no generally accepted metric to determine how much deviation is accepted for a model to still be considered to be valid. The jaggedness, with large steps in the error rate, of (A), is due to the smaller training set (only using cohort 1), compared to (B) (using both cohorts as training data).

Table S1. Exact p-values for individual metabolites, related to Table 3. Exact p-values for each of the selected metabolites, from Table 3, where only significance levels are given.

m/z (Da)	RT (sec)	FC PMS-RRMS (cohort 1)	p-value PMS-RRMS (cohort 1)	sex p-value (cohort 1)	FC PMS-RRMS (cohort 2)	p-value PMS-RRMS (cohort 2)	sex p-value (cohort 2)
215.048564544976	109.693521060547	0.6	0.000152748	0.072269486	0.74	0.000166163	0.668155591
272.185644335785	407.111169610083	0.38	6.26E-05	0.005687554	0.37	0.005365194	0.506538726
128.890701122674	37.6567891874419	-0.19	0.001262994	0.099963775	-0.01	0.795014924	0.852602849
146.081094191808	73.0755488204299	0.26	0.000686591	0.40676297	0.4	0.00068617	0.205342286
316.248367808529	591.523063746979	0.57	0.000155596	0.239068782	0.1	0.28263638	0.552249544
278.102329619794	309.849981251338	0.28	0.000337293	0.744096921	0.49	0.00014234	0.739972484
264.207115715347	358.927356617333	0.64	0.000879244	0.770868858	0.48	0.005159018	0.90933998
295.165404856152	352.197761045332	0.48	0.00436032	0.228185851	0.1	0.386020016	0.12913574
182.067353328031	80.6216817099774	0.36	0.002229933	0.135999683	0.47	0.007490807	0.606400831
158.117729372469	372.810643000107	0.54	0.007697124	0.648503154	0.76	0.003195327	0.048724309
234.206528982132	487.140733014978	0.46	0.007865705	0.620092395	-0.02	0.929071213	0.806111094
374.164085940078	619.382879937271	-0.18	0.015823483	0.699969316	-0.13	0.08988273	0.03234101
393.220082646112	829.240507816282	0.16	0.006992957	0.204019557	0.03	0.472187483	0.614111482
123.055474504947	58.3232544699935	0.59	0.00991863	0.111171077	0.01	0.97385751	0.324616488
127.050360653391	67.359161989063	0.19	0.029909073	0.660471994	0.51	0.000548114	0.349723814

Table S2. Settings for metabolite quantification, related to STAR Methods. Non-default parameter values used for pre-processing in KNIME. For all parameters not mentioned, the default values were used. Intermediate headers in italics are the two R packages that were used.

Parameter	Value
FeatureFinderMetabo	
noise_threshold_int	100
chrom_peak_snr	2.0
chrom_fwhm	3.0
mass_error_ppm	5.0
min_trace_length	3.0
width_filtering	auto
report_convex_hulls	true
FeatureLinkerUnlabeledQT	
threads	30
no_progress	true
nr_partitions	10
ignore_charge	true
max_difference (distance_RT)	10.0
max_difference (distance_MZ)	5.0
unit	ppm

Table S3. Conformal predictions of transitioning patients, related to Figure 2. Conformal p-values (p) of the RRMS and PMS phenotypes for the eight transitioning patients. Patients that would be given a single-label prediction of PMS when utilizing a significance threshold of 6% have been highlighted in *red*, whereas patients with single-label predictions of RRMS have been highlighted in *blue*. Patients who would receive a double-label prediction have been marked in *grey*. Conformal p-values higher than the hypothetical significance threshold of 6% have been bolded. Three of the transitioning patients are predicted as PMS with single-label predictions (T1-T3), four as RRMS with single-label predictions (T5-T8) and one patient (T4) would receive a double-label prediction.

Patient	T1	T2	T3	T4	T5	T6	T7	T8
**p[RRMS]**	0.034	0.041	0.051	**0.082**	**0.168**	**0.194**	**0.320**	**0.958**
**p[PMS]**	**0.933**	**0.639**	**0.579**	**0.104**	0.051	0.029	0.005	0.011

Table S4. Conformal predictions of ITT-PMS patients, related to Figure 5. Conformal p-values (p) of the participants in the ITT-PMS trial at baseline (before treatment), and at their three-, six-, and twelve-months follow-up. A sample from patient 6 at the three months follow-up was missing. Patients that would be given a single-label prediction of PMS when utilising a significance threshold of 5% have been highlighted in *red*, whereas patients with single-label predictions of RRMS have been highlighted in *blue*. Patients who would receive a double-label prediction have been marked in *grey*. Conformal p-values higher than the hypothetical significance threshold of 5% have been bolded.

	**Baseline**		**3 months**		**6 months**		**12 months**	
	p[RRMS]	p[PMS]	p[RRMS]	p[PMS]	p[RRMS]	p[PMS]	p[RRMS]	p[PMS]
**Patient 1**	0.0114	**0.9142**	0.0275	**0.5432**	**0.1725**	**0.5210**	**0.2474**	**0.3192**
**Patient 2**	0.0298	**0.5209**	**0.2178**	**0.1715**	**0.2506**	**0.0989**	**0.2093**	**0.3278**
**Patient 3**	0.0197	**0.6159**	0.0315	**0.8740**	0.0011	**0.9468**	0.0122	**0.8402**
**Patient 4**	0.0101	**0.9719**	0.0067	**0.9376**	0.0009	**0.9789**	0.0010	**0.9462**
**Patient 5**	**0.1718**	**0.4725**	**0.1727**	**0.2533**	**0.1814**	**0.0932**	**0.1405**	**0.2993**
**Patient 6**	**0.2526**	**0.0646**	-	-	**0.2572**	**0.0780**	**0.1844**	**0.0988**
**Patient 7**	0.0065	**0.8851**	0.0054	**0.9218**	0.0451	**0.5075**	0.0014	**0.9678**
**Patient 8**	0.0146	**0.8442**	0.0108	**0.6494**	**0.2030**	**0.3360**	**0.1987**	**0.2668**
**Patient 9**	**0.6079**	0.0012	**0.4599**	0.0228	**0.4321**	0.0020	**0.4187**	0.0063
**Patient 10**	**0.4107**	**0.3000**	**0.3859**	0.0283	**0.2847**	**0.0579**	**0.2548**	**0.3723**
**Patient 11**	0.0026	**0.7597**	0.0078	**0.7192**	0.0157	**0.6689**	0.0126	**0.7211**
**Patient 12**	0.0377	**0.4941**	0.0385	**0.3971**	**0.1410**	**0.3904**	**0.2246**	**0.3263**
**Patient 13**	0.0282	**0.6515**	0.0310	**0.6525**	0.0079	**0.8094**	0.0360	**0.6577**
**Patient 14**	**0.2382**	**0.2883**	**0.1440**	**0.3194**	**0.1239**	**0.1735**	**0.3245**	0.0459
**Patient 15**	**0.0669**	**0.2896**	**0.3834**	0.0212	**0.2697**	**0.0988**	**0.1364**	**0.0875**
**Patient 16**	0.0315	**0.5755**	0.0182	**0.4974**	0.0105	**0.5931**	0.0344	**0.3585**
**Patient 17**	0.0025	**0.8165**	**0.2322**	**0.2812**	**0.1748**	**0.3041**	0.0031	**0.7557**
**Patient 18**	**0.0530**	**0.4070**	**0.2311**	**0.1865**	0.0141	**0.8337**	**0.2256**	**0.3082**
**Patient 19**	<0.0001	**0.6943**	0.0024	**0.9582**	0.0077	**0.8586**	0.0007	**0.7419**
**Patient 20**	**0.2135**	**0.1058**	**0.3307**	**0.0570**	**0.4908**	**0.0853**	**0.4059**	0.0382
**Patient 21**	0.0122	**0.7810**	0.0138	**0.8349**	**0.0831**	**0.7376**	**0.1005**	**0.3971**
**Patient 22**	0.0267	**0.4333**	0.0243	**0.9895**	0.0359	**0.5548**	0.0479	**0.4018**
